# Editorial: Anesthetic neurotoxicity in developing brains: mechanisms, biomarkers, and therapeutic targets

**DOI:** 10.3389/fneur.2023.1279529

**Published:** 2023-08-31

**Authors:** Yang Yu, Yonghao Yu, Yiying Zhang, Huihui Miao

**Affiliations:** ^1^Department of Anesthesia, Tianjin Medical University General Hospital, Tianjin, China; ^2^Tianjin Institute of Anesthesiology, Tianjin, China; ^3^Department of Anesthesia, Critical Care and Pain Medicine, Massachusetts General Hospital and Harvard Medical School, Charlestown, MA, United States; ^4^Department of Anesthesia, Beijing Shijitan Hospital, Capital Medical University, Beijing, China

**Keywords:** general anesthetics, neurotoxicity, neurocognitive impairment, biomarkers, therapeutic targets, developing brain

As editors of this Research Topic, it was our delight to gather a diverse variety of intriguing papers and reviews to advance our understanding of the age-dependent effects of anesthetics on developing brains and the underlying mechanisms. In this editorial we recapitulate the major findings and perspectives detailed.

The Research Topic covers eight articles, comprising four original research studies and four reviews, with contributions from 47 authors from three countries. This Research Topic spans several issues, including the mechanisms underlying neurotoxicity and cognitive impairments induced by general anesthetics in neonates, which involve numerous molecular and cellular-level mechanisms. It evaluates the important focal topics and present state of clinical and basic research in this sector separately. Notably, transferring information from animal investigations into clinical treatment poses significant challenges. The components of clinical research encompass exploring the effects of prenatal anesthesia exposure on neonates and investigating the impact of using anesthesia monitoring devices during the perioperative period. Furthermore, this topic includes applicable neuroprotective techniques as well as a variety of prospective therapeutic intervention approaches. Within this Research Topic, eight articles provide varied viewpoints and ideas that improve our understanding of the area. The primary objective is to give unique insights into maintaining neonatal nervous system health and development throughout the perioperative period, while also providing thorough support for clinical applications.

Growing evidence suggests that early-life general anesthetic exposure causes nerve cell death, which might lead to cognitive impairment (1, 2). The underlying mechanics are complicated. Wang and Liu presented an overview of the molecular signaling pathways behind anesthesia-induced neonatal postoperative neurotoxicity and cognitive impairment. They studied routes such as the HIPK2/Akt/mTOR signaling pathway, the PI3K/Akt signaling pathway, and the HIPK2/JNKs/c-Jun signaling pathway, among others, in order to provide vital insights for the treatment of clinical disorders. Sevoflurane is the most often used general anesthetic in pediatric surgical operations, yet, it has the potential for neurotoxicity (2, 3). Feng et al. employed a combined quantitative proteomic technique using TMTpro tagging and LC-MS/MS to examine 6,247 proteins, 443 of which were involved with the age-dependent neurotoxic processes generated by multiple sevoflurane anesthesia. Furthermore, studies using western blotting demonstrated that sevoflurane-induced brain injury in newborn mice may be mediated by raising the levels of protein expression of CHGB, PTEN, MAP2c, or reducing the level of SOD2 protein expression. Li et al. reported that newborn mice exposed to sevoflurane for an extended period of time had hyper-ramification of microglia. This exposure reduced microglia-synapse interactions in neonates without affecting their surveillance or response to brain injury. These microglial changes linked to adult anxiety-like behaviors. Pre-exposure microglial depletion and subsequent re-population lessened sevoflurane-induced anxiety. Microglia may represent a significant target for developmental neurotoxicity associated with general anesthetics. Increasing evidence suggests that mitochondria are potential primary targets of anesthetic-induced injury (4, 5). Mitochondrial dependency is further emphasized in the growing brain, which necessitates spatiotemporally complicated and metabolically costly processes such as neurogenesis, synaptogenesis, and synaptic pruning, implying that functional modifications might have a significant influence. To this end, Hogarth et al. investigated and reviewed existing knowledge of processes behind the influence of anesthetic exposure on mitochondria in the developing nervous system. They concentrated on the effects of anesthetics on mitochondrial dynamics, cellular apoptosis, bioenergetics, stress pathways, and redox homeostasis in particular. They also identified critical information gaps, related obstacles, and prospective treatment targets that should be investigated further in order to drive mechanistic and outcomes research.

Animal model research undertaken in the early 21^st^ century revealed that general anesthetics can cause neuro-apoptosis and impair neuronal development in animals ranging from rodents to non-human primates. However, putting this data into clinical practice has been difficult. Nonetheless, several clinical studies show that when infants and toddlers are treated to systemic anesthesia during operations, they are at an increased risk of postoperative neurotoxicity and cognitive impairment, particularly during important phases of brain development (1, 6). Managing and mitigating such risks constitute pivotal concerns for clinicians. Identifying neuroprotective compounds capable of mitigating toxicity is imperative. Dexmedetomidine emerges as a potential neuroprotective agent that may circumvent the neurotoxicity potentially associated with anesthesia. Tsivitis et al. provided a comprehensive review of the application of dexmedetomidine in anesthesia. Dexmedetomidine is a sedative and analgesic having agonist actions on alpha-2 (α2) adrenoceptors as well as imidazoline type 2 (I2) receptors, allowing it to impact intracellular signaling and modify cellular processes. Numerous animal studies show that dexmedetomidine protects neurons from apoptosis, ischemia, and inflammation while sustaining neuronal plasticity. Furthermore, several researchers have focused on the use of specific anesthetic monitoring equipment during the perioperative phase. Despite the fact that bispectral index (BIS)-guided total intravenous anesthesia (TIVA) is routinely used in pediatric settings, few studies have been conducted to evaluate its role in younger children. Liu et al. designed a prospective, randomized, single-blind, controlled clinical experiment to assess the efficacy of BIS-guided TIVA in pediatric anesthesia. Their research found that using BIS-guided pediatric TIVA did not reduce extubation time or post-anesthesia care unit (PACU) stay duration.

Preclinical animal researches have shown that intrauterine anesthesia causes newborn neurotoxicity, which is shown mostly by tissue morphological abnormalities and impairments in learning and memory capacities (3, 7, 8). The long-term effects of prenatal anesthetic exposure (PAE) on neurodevelopment remain unknown. Zhou et al. summarized the advancements in understanding the outcomes of prenatal anesthesia exposure on offspring neurodevelopment through a combination of animal experiments and clinical research. Additionally, they provided a concise overview of childhood neurodevelopment and potential confounding factors.

Cao et al. aimed to comprehensively outline the current status of this field by investigating the research trends and publication patterns concerning anesthetic-induced neurotoxicity in brain development. Research indicates that clinical studies in this field primarily consist of retrospective investigations, underscoring the need for heightened emphasis on prospective, multicenter, and long-term monitoring clinical studies in the future. The article also highlights the ongoing need for extensive fundamental research to elucidate the mechanisms of anesthetic-induced neurotoxicity in brain development.

In conclusion, the current investigations shed light on progress in anesthetic-induced neurotoxicity and cognitive impairments in developing brain, as well as its future challenges. We hope that the information gathered from this Research Topic will inspire, update and provide guidance to researchers in the field.

## Author contributions

YaY: Investigation, Project administration, Writing—original draft. YoY: Writing—original draft. YZ: Writing—review and editing. HM: Writing—review and editing.
